# Synthetic mimetics of protein secondary structure domains

**DOI:** 10.1098/rsta.2009.0210

**Published:** 2010-03-13

**Authors:** Nathan T. Ross, William P. Katt, Andrew D. Hamilton

**Affiliations:** Department of Chemistry, Yale University, PO Box 20810, New Haven, CT 06520, USA

**Keywords:** protein mimetics, α-helix mimetics, stapled peptides, β-peptides, terphenyl

## Abstract

Proteins modulate the majority of all biological functions and are primarily composed of highly organized secondary structural elements such as helices, turns and sheets. Many of these functions are affected by a small number of key protein–protein contacts, often involving one or more of these well-defined structural elements. Given the ubiquitous nature of these protein recognition domains, their mimicry by peptidic and non-peptidic scaffolds has become a major focus of contemporary research. This review examines several key advances in secondary structure mimicry over the past several years, particularly focusing upon scaffolds that show not only promising projection of functional groups, but also a proven effect in biological systems.

## 1. Introduction

One of the fundamental tenets of biology is that protein structure dictates protein function. Within the context of chemical biology, small molecule mimetics of protein structures are often sought to recapitulate key protein contacts while taking advantage of the chemical properties inherent to synthetic molecules. Furthermore, these structural mimetics must be capable of modulating the interaction between their proteinaceous predecessor and its natural binding partner. In this sense, just as protein structure dictates protein function, the ability of the synthetic molecule to mimic a given protein structure will directly affect its efficacy in a biological setting.

The predominant manner by which protein functions are controlled in nature is via protein–protein interactions. These interactions cause the burial of a large, previously solvent-exposed surface that averages 1600±400 Å in size (Cunningham & Wells 1989; Janin & Chothia 1990). These interactions are governed by hydrophobic, hydrogen bonding, ionic and van der Waals contacts between the two protein surfaces. While mimicry of these large surfaces with synthetic molecules is challenging, the task has been simplified by the discovery of protein hot-spots; areas or groups of residues that confer the majority of favourable free energy for protein–protein association (Cunningham & Wells 1989). Highly ordered secondary structure motifs, such as the α-helix, β- and γ-turns, and β-sheets, have all been observed to be critical display scaffolds for key amino acid residues in protein–protein hot-spots.

A highly active field has emerged involving the design of small molecules that mimic these ordered secondary structure domains and display functionality in a similar fashion. This review examines several of the most important recent advances in this arena, focusing particularly upon those that show not only the ability to mimic protein secondary structure, but also to modulate protein function in a biological system.

## 2. α-Helices

The α-helix is the most common secondary structure encountered in proteins (Pace & Scholtz 1998). As a result, it has been a major target for small molecule mimicry. Mimetics range from slight derivations of natural peptides to small molecules that bear little resemblance to an α-helix. Early mimetic structures suffered from poor pharmacokinetic properties and often lacklustre binding affinities; accordingly, recent efforts have been directed at resolving these issues.

### (a) Peptoids

One of the most simple and effective means of altering the properties of a peptide and encouraging a helical structure is to convert it to an analogous poly-*N*-substituted glycine, or peptoid (Zuckermann *et al.* 1992). Peptoids, unlike peptides, have side chains linked directly to their nitrogen atoms, rather than to their α-carbon atoms. While this eliminates the inherent chirality found in peptides, and thus would seemingly negate the possibility of helix formation, α-helix like structures have been induced via the use of chiral *N* side chains. Considerable work on structured peptoids has been reported (Stigers *et al.* 1999; Patch & Barron 2002). Several recent key contributions show the potential value of peptoids as antimicrobial agents, as an artificial lung surfactant and as mimics of higher order secondary structure.

**Figure 1.**
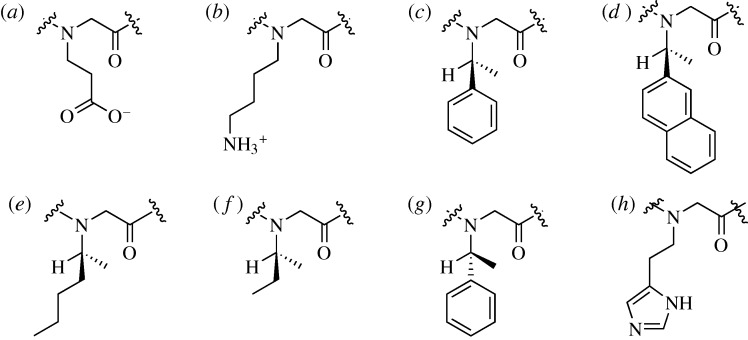
*N*-substituted glycine monomers used for the construction of helical peptoids by the Barron laboratory. (*a*) *N*Glu, (*b*) *N*Lys, (*c*) *N*Spe, (*d*) *N*sna, (*e*) *N*smb, (*f*) *N*ssb, (*g*) *N*rpe and (*h*) *N*His.

Barron and co-workers have recently shown the potency of peptoids as antimicrobial agents (Chongsiriwatana *et al.* 2008). While antimicrobial peptides are well known and highly potent agents, they suffer from low bioavailability owing to the short half-life imposed by protease degradation. Peptoids avoid this problem owing to the masking of their amide bond, and subsequent inability to tightly bind proteases (Miller *et al.* 1994). Barron constructed peptoids out of a small library of peptoid monomers (figure 1). These monomers were tuned primarily for hydrophobicity, with only one monomer each having a positively or negatively charged side chain. Compared with two antimicrobial peptides, pexiganan (Ge *et al.* 1999) and melittin (Fennell *et al.* 1968), with respective minimum inhibitory concentrations (MICs) versus *Escherichia coli* of 3.1 and 1.6 μM, and selectivity ratios (defined as the 10% haemolytic dose divided by the *E. coli* MIC) of 24 and 1.3, prepared peptoids had MICs versus *E. coli* between 3 and 30 μM and selectivity ratios between 1 and 20. Hence, peptoid compounds, depending upon exact composition, showed similar potency and cytotoxicity to natural analogues. The overall hydrophobicity and net charge of these peptoids were found to have a much greater bearing on their biological effects than the exact side chains chosen, with more hydrophobic compounds generally being more potent and less selective, while compounds with a net positive charge had slightly lower activity but showed greatly reduced cytotoxicity.

In addition to antimicrobials, Barron has also adapted peptoids to function as analogues of SP-C, a component of lung surfactant, the biomaterial found at the air–liquid interface of lungs (Brown *et al.* 2008). Treatment of lung surfactant maladies currently involves replacement of surfactant with animal-lung-derived compounds (Notter 2000). This can lead to numerous health complications, such as poor antigen transfer and supply problems, making a totally synthetic solution highly desirable. Peptoids were prepared from the same pool of monomers as those designed as antimicrobial agents. Studies showed that the ability of peptoids to mimic SP-C was derived primarily from the degree of helicity and overall hydrophobicity, either of which had a much greater impact upon the biological activity of the peptoid than did the exact side chains chosen at any given position. The length of the peptoid helical regions was also found to play an important role, with a peptoid having a 28 Å long helix being best able to anchor in the lipid film of lung surfactant. Interestingly, these studies both suggest that peptoids, while able to mimic the general shape of peptides, may not make the same interactions with the target, as side-chain selection should bear much more influence over observed biological activity.

**Figure 2.**
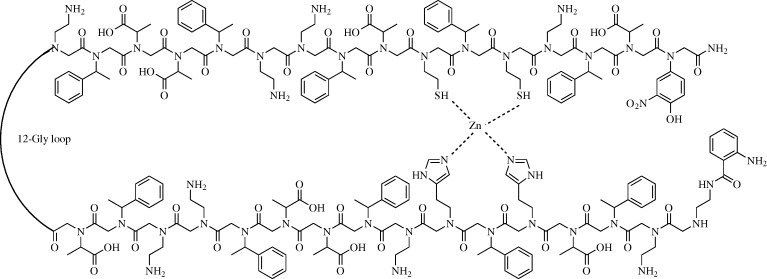
Cysteine and histidine mimetic peptoids bind to a zinc atom, bringing two helices into a bundle.

Zuckermann has taken a different approach to highly structured peptoid formulation. Rather than attempting to target an α-helix binding site, he mimicked larger super-structure motifs found in proteins (Lee *et al.* 2008). Recently, he adapted a two-helix peptoid bundle, with helices linked by a poly-glycine loop, to bind zinc atoms. Using very simple helices, but adding thiol and imidazole groups at select positions, Zuckermann was able to bind zinc atoms with apparent *K*_d_ values as low as 0.3 nM (figure 2).

The length of the linker between the two helices was found only to matter when it was too short to allow optimal zinc coordination. These results demonstrated that peptoids can be used not only to mimic α-helices, but also to mimic higher order helical systems. However, most peptoid research aims to mimic only random coil peptides, not peptides with well-defined secondary structures, leaving the arena wide open for further developments.

### (b) *β*-Peptides

Another important class of biomimetics that can be made to adopt a helical conformation are β-peptides. These structures are generally composed of β^3^-amino acids and adopt a 14-helical structure under most conditions, although they can also be tailored to form 10/12 or 12 helicies (Kritzer *et al.* 2005). In addition, owing to their non-standard backbone sequence, they are resistant to most proteases that degrade common α-peptides (Frackenpohl *et al.* 2001). While early research into β-peptides revolved around synthesis and preliminary biological evaluation (Cheng *et al.* 2001), recent work has focused on higher order β-peptide assembly and more complex biological applications.

**Figure 3.**
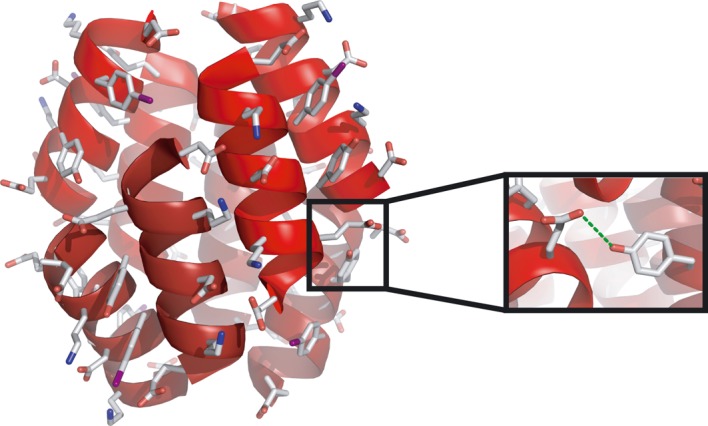
Structural insights into the assembly of β-peptides can be gained from X-ray crystal structures depicting the three-dimensional structure of fully assembled β-peptides such as this one from Goodman *et al.* (2007).

The Schepartz group has studied how β-peptides assemble in aqueous solution and how the residue composition can dictate their higher order assembly into protein-like macromolecular bundles. The first X-ray crystal structure of a β-peptide bundle showed the role of the hydrophobic edge of the β-peptides in clustering at the core of an octomeric bundle (Daniels *et al.* 2007). More recently, a second structure has been published also displaying an octomeric overall structure (Goodman *et al.* 2007). In this case, the hydrophobic core was maintained, but in addition a network of intramolecular hydrogen bonds was proposed on the exterior of the octomeric bundle (figure 3). This work also detailed a thorough biophysical analysis of these bundles, confirming their octomeric structures in solution. In addition, Schepartz has been able to regulate the tertiary assembly of these bundles by varying the side-chain composition. In one report, a smaller tetrameric bundle was generated (Goodman *et al.* 2008), whereas in a second a previously octameric bundle was re-engineered into a tetramer using a β^3^-glycine–β^3^-glycine linker (Petersson & Schepartz 2008). These higher order bundles all have molecular weights of the order of 10 kDa, making them viable for consideration as full-size protein mimetics.

To further probe the biological efficacy of β-peptide-based structures the Schepartz and Gellman groups have both pursued a number of important biological targets. Schepartz has effectively shown that a β-peptide can mimic p53 and bind to hDM2 in the p53 binding cleft (Kritzer *et al.* 2004). In a second study, they have also shown that β-peptides can be rationally designed to target gp41 and inhibit HIV-1 cell fusion (Stephens *et al.* 2005). Gellman and co-workers have also established biological activity in their β-peptides, targeting both human cytomegalovirus (HCMV) entry (English *et al.* 2006) and herpes simplex virus type-1 (HSV) (Akkarawongsa *et al.* 2008). In preventing HCMV, β-peptides were designed to mimic the gB glycoprotein heptad repeat (HR) and block cell fusion in a similar mechanism to those designed by Schepartz for HIV-1. However, those targeting HSV are cationic, cell permeable, and appear to impose their anti-HSV effect at the post-attachment step in HSV infection.

### (c) Stapled peptides

Slight modifications of normal peptide sequences can have profound effects upon their ability to accurately mimic various secondary structure elements. One example of this effect is seen in the so-called peptide stapling. In many cases, short peptide sequences, even those found in the middle of a highly stable α-helix in a protein, are unable to achieve a helical conformation alone (Baldwin 1995). This is presumably due to entropic penalties, as a disordered chain of residues is entropically favoured over a well-ordered chain (Creamer & Rose 1992; Aurora *et al.* 1997). Peptide stapling overcomes this problem by covalently linking residues which should be on the same face of a helix, reducing the maximum length between the two and making helical formation more likely. Recent work by the Walensky, Cowburn, Verdine and Fairlie groups has shown how promising this technique can be by preparing stapled peptides active versus the Bcl-2 family of proteins, the HIV-1 capsid protein, hDM2 and respiratory syncytial virus (RSV).

**Figure 4.**
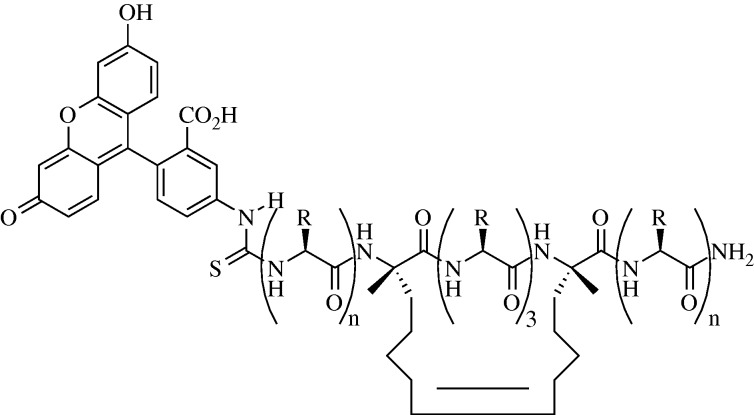
Walensky stapled peptide, which had increased affinity for Bcl-2 over comparable unstapled compounds.

Bcl-2 proteins are a family of apoptosis governors, key regulators of the cell cycle. The Bcl-2 homology domain 3 (BH3) is a helical region that activates pro-apoptotic functions of Bcl-2 members. Walensky has designed mimics of several such helices, each stabilized via addition of an olefin-capped propyl chain to each of two residues not involved in Bcl-2 binding, followed by subsequent ruthenium-catalysed olefin metathesis (figure 4). These compounds were tested for binding affinity towards Bcl-2 proteins via numerous methods, including fluorescence polarization (testing direct binding of the helix to a target protein), liposomal release assay (which models a simplified mitochondrial system) and a mitochondrial cytochrome C release assay (in which mitochondria are isolated from other cellular material and assayed). In each case, the stabilized version of a peptide showed superior binding compared with the unstabilized analogue (Bird *et al.* 2008).

In addition, Walensky showed that stabilized helices exhibited favourable biophysical properties. Circular dichroism established that ‘stapled’ peptides did exhibit more helical character (approx. 80% helicity) than unlinked peptides (approx. 15% helicity). Owing to the forced α-helix conformation, the peptide chains had fewer exposed peptide bonds and showed resiliency against proteases. Interestingly, stabilized helices were found to exhibit increased cell permeability, possibly owing to the helical nature of the peptides (similar to transmembrane proteins) and the hydrophobic character of the hydrocarbon staples (Pitter *et al.* 2008).

**Figure 5.**
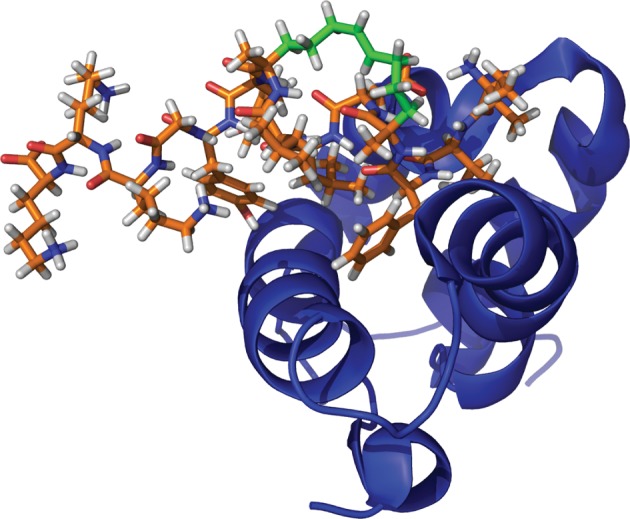
NMR solution structure of a stapled peptide (orange) bound to capsid (blue). The staple region (green) does not interact with the protein binding site.

Stapled peptides have been targeted to other proteins, such as HIV-1 capsid, a symmetric dimeric protein which plays a vital role in the formation of HIV viral particles (Bhattacharya *et al.* 2008). Cowburn’s NYAD-1, a stapled 12-mer peptide, exhibited a *K*_d_ of 10 μM versus capsid. In order to increase aqueous solubility, NYAD-13 was developed, in which a *C*-terminal proline was replaced with three lysine residues. This increased solubility allowed the determination of the NMR solution structure of the peptide and protein (figure 5). Though the interaction between the stapled peptide and protein target is composed primarily of hydrophobic contacts, the hydrophobic staple is not involved in binding, but unfavourably exposed to solvent.

Verdine recently used stapled peptides to target the p53/hDM2 interaction, an important regulatory element in the apoptotic pathway governed by a 15-mer helical segment of p53 (Bernal *et al.* 2007). In an initial library derived from the p53 wild-type sequence, binding affinities for hDM2 as low as 0.9 nM were observed. However, members of this original series were negatively charged and unable to penetrate Jurkat T-cells. To effect cell penetration, new stapled peptides with positive charges were prepared. While some potency was lost, the best compound still exhibited 55 nM binding affinity, was cell permeable and importantly was able to restore apoptotic activity in compromised cells, unlike the wild-type peptide.

**Figure 6.**
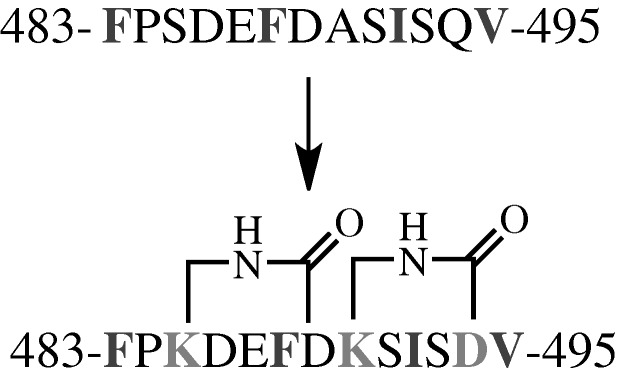
Peptide sequences used by Shepherd *et al.* (2006). Key interacting residues are indicated in dark grey, mutated residues that allowed for lactam constraints are light grey.

In another use of staple-based mimetics, the Fairlie group has developed an interesting, rationally designed, bi-cyclic peptide that is capable of adopting a highly α-helical structure in aqueous solution and disrupting RSV cell fusion (Shepherd *et al.* 2006). This 13-mer is made of up two five-residue cyclic rings with three linear peripheral residues and is based upon a portion of the HR *C*-terminal (HR-*C*) helix of the RSV F glycoprotein. This surface protein is a trimer of F1 and F2 dimer units. The F1 portion contains an α-helical HR-*C* and HR-*N*-terminal region. In order for cell fusion to occur, the HR-*C* region must undergo a conformational change and fold back upon the HR-*N* region of F1. The design principle of this research was to mimic a portion of the HR-*C* and inhibit binding to HR-*N*, which was predicted to affect viral cell fusion. Original peptide designs were based directly upon the sequence of HR-*C* that was known to interact with HR-*N* from the crystal structure of the post-conformational change form of F1 (Shepherd *et al.* 2006). A 13-residue core sequence was selected (483-FPSDEFDASISQV-495), of which residues F483, F488, I492 and V495 were observed to interact directly with HR-*N* (figure 6). To constrain this sequence, Fairlie and co-workers looked to the solvent-exposed residues and made selected point mutations, S485K, A490K, Q494D. This mutant peptide could then be constrained by making two K(i) to D(i+4) lactam bridges, and was found to be highly α-helical by circular dichroism. This constrained bi-cyclic peptide has a root mean square deviation (RMSD) overlay of backbone atoms of HR-*C* of 0.99 Å, and was found to inhibit cell fusion with low nanomolar IC_50_ values. The peptide also had antiviral activity against RSV, which reduced RSV plaque number in a dose-dependent fashion, with an IC_50_ of 36 nM. This activity is much greater than the full length HR-*C* peptide, which had been previously shown to reduce RSV plaques with an IC_50_ of 2.93 μM.

These studies have shown the power of peptide stapling as a method to turn simple peptides into well-folded, biologically active compounds. As the approach develops, it is likely that the staples themselves may be integrated into binding schemes and become more than mere structure modulators.

### (d) Small molecules and assemblies

**Figure 7.**
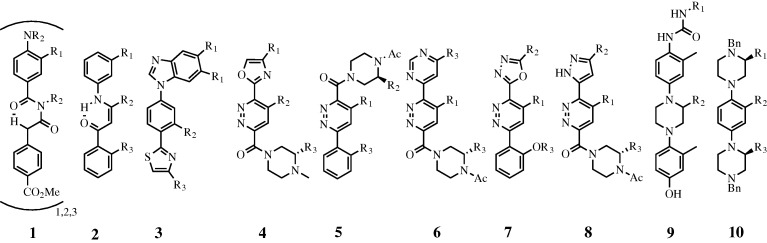
Recent α-helix mimetics from the Hamilton (**1**–**3**), Rebek (**4**–**9**) and Konig (**10**) groups.

Apart from the generally peptidic structures above, numerous non-peptidic small molecules have been developed to mimic α-helicies. Early contributions from Hamilton and co-workers showed that a terphenyl scaffold was capable of projecting functionality in a similar manner to the i, i+3 (or i+4) and i+7 residues of an α-helix (Orner *et al.* 2001). Since then, efforts have been exerted to design similar teraryl molecules, and several reviews on these molecules have been published (Yin & Hamilton 2005; Davis *et al.* 2007). In light of this, we only aim to cover structures that have been reported within the last two years. A driving force in the recent literature has been the desire to decrease overall compound hydrophobicity as well as reduce the synthetic complexity of teraryl-based α-helix mimetics. This has been accomplished either by increasing the heteroaromatic nature of the core aryl units (figure 7, compounds **3**–**10**) or by replacing some of the covalent character of the scaffold with a hydrogen bonding aromatic ring isostere (figure 7, compounds **1**, **2**).

The structure of a 5-6-5 imidazole-phenyl-thiazole scaffold (**3**) (Cummings *et al.* 2009) bearing additional heteroaromatic functionality has recently been reported. Rebek and co-workers have also published a series of pyridazine-based scaffolds (**4**–**8**) with a variety of aromatic and heteroaromatic peripheral groups (Biros *et al.* 2007; Volonterio *et al.* 2007). Additionally, two piperazine based scaffolds (**9**, **10**) have been reported, one by Konig (**10**) incorporating two peripheral piperazine units with a central benzene core (Maity & Konig 2008), and a second by Rebek (**9**), with one central piperazine unit with peripheral benzene units (Restorp & Rebek 2008). Both display functional groups in a manner to mimic three or four α-helicial side-chain positions. We have recently shown that hydrogen bonding functional groups such as an enaminone (**2**) (Rodriguez & Hamilton 2006) or benzoylurea (**1**) (Rodriguez & Hamilton 2007) can replace a central six-membered ring, reducing hydrophobicity and synthetic complexity of these types of scaffolds.

Another noteworthy advance has been the incorporation of stereocentres into the teraryl-based α-helix mimetics. The original Hamilton terphenyl molecules, while mimicking a chiral α-helix, are themselves achiral. However, Rebek and Konig have now incorporated one and two stereocentres, respectively, into their mimetics (**4**–**8**, **10**). While potentially adding to the synthetic complexity of helical mimetics, the presence of stereogenic chiral centres may eventually be important in protein binding studies.

In later work, we have addressed elongation of these scaffolds to the mimicry of three or four side-chain positions, as well as addressing new biological targets beyond Bcl-xL/Bak and p53/MDM2. We have recently shown that a benzoylurea scaffold (**1**) can be elongated to mimic as many as nine side groups of an α-helix; a compound that when fully elongated spans almost 40 Å (Rodriguez & Hamilton 2007)

**Figure 8.**
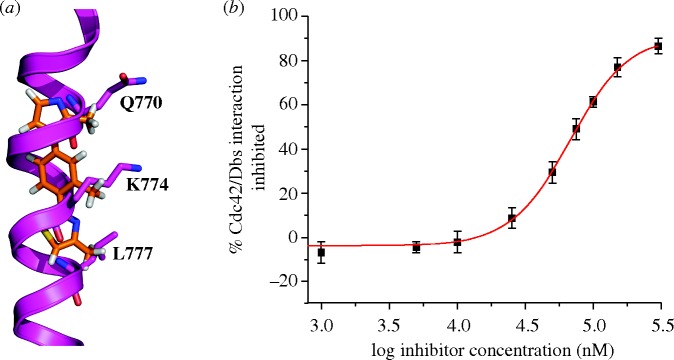
(*a*) Overlay of the X-ray crystal structure of a trismethyl 5-6-5 imidazole-phenyl-thiazole derivative with key contact residues from Dbs. (*b*) Inhibition data of 5-6-5 compound, which displayed a 67 μM IC_50_ value against the Cdc42/Dbs interaction as measured by the ability to disrupt Dbs assisted uptake of mantGDP by Cdc42.

Using the 5-6-5 imidazole-phenyl-thiazole scaffold (**3**), we have generated the first rationally designed, small molecule inhibitor of the interaction between the GTPase Cdc42 and its associated guanine nucleotide exchange factor Dbs (Cummings *et al.* 2009) with a 67 μM IC_50_ (figure 8). Like previous helix mimetics, these were designed to mimic the i, i+4 and i+7 positions of a target α-helix; in this case, Q770, K774 and L777 of Dbs. However, when compared with earlier generations of mimetics, these possess significantly increased solubilities. The calculated log *P* (o/w) values of trimethyl-substituted terphenyl and terpyridine compounds are 7.3 and 3.4, respectively, while that of the trimethyl 5-6-5 imidazole-thiazole-phenyl compound is 2.3.

Although most biomimetic efforts have been devoted to the α-helix, this is not the only type of helical molecule found in nature. Several types of helices, including α-helices, 3_10_ helices, pi helices and double helices are found in peptides and DNA. Likewise, other areas of chemistry have found a use for modified helices. One such area has been investigated by Pepitone, who used a supramolecular helical scaffold to model photosynthetic properties (Kim *et al.* 2008). In nature, chlorophyll molecules are often used as light harvesting antennae, and are arranged in circular formations about photoreactive centres. This arrangement has been modelled by a helical scaffold wrapped around a central, rod-like photoacceptor core.

**Figure 9.**
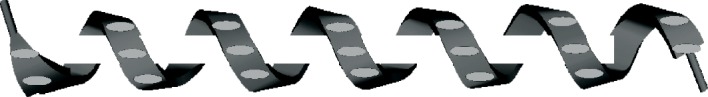
A superhelix antenna array. A DASP chromophore (white rod) is encapsulated by a helix (dark grey) with the assistance of CMA aggregates (light grey ovals).

Pepitone used polycarboxymethyl amylose (CMA) with 6 per cent to 100 per cent of amylose units carboxymethylated (the DS value of the polymer). While CMA did not form a helical structure alone, addition of one of three dyes, a cyanine-based light harvesting dye or a rodlike acceptor dye (4-(4-(dimethylamino)styryl)-1-alkylpyridinium bromide (DASP)-C22 or DASP-6V12), led to helix formation. Subsequent addition of the complementary dye completed the antenna ensemble (figure 9). Regardless of the order of dye addition to the CMA polymer, the acceptor was wrapped on the inside of the helix and the cyanine dye studded the helix’s surface.

These superhelices act as excellent electron transfer systems. A quenching efficiency of greater than 90 per cent was observed when the cyanine dye molecules are excited, which compares well with the nearly 100 per cent efficiency in natural photosynthetic systems. Similarly, the fluorescence lifetime of the donor was seen to decrease fivefold when placed in this system, suggesting rapid and stable quenching by the DASP dyes. These two properties suggest that the antenna system works as an effective system for artificial photosynthesis.

## 3. Turn structures

In addition to α-helices, organized turn motifs are among the most well-studied secondary structural features of proteins. These turns generally involve three to five amino acids, and impose well-defined phi and psi angles not otherwise available to helices, sheets or extended peptide sequences. Seminal work from the Robinson (Robinson 2008; Robinson *et al.* 2008), Mayo (Dings & Mayo 2007) and Nowick (2008) laboratories on turn mimicry has recently been reviewed.

**Figure 10.**
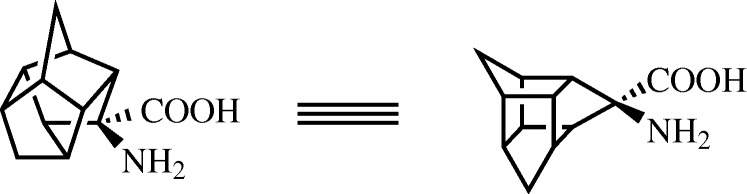
Structure of the trishomocubane amino acid used by Albericio *et al.* (2008) to generate a β-turn structure with three amino acids on either side of the turn inducing cubane.

An intriguing example of a fully synthetic turn inducing scaffold comes from Albericio *et al.* (2008), who described the synthesis of a novel trishomocubane amino acid (figure 10) used in the centre of the hexa-alanine polypeptide to generate a (Ala)_3_-cage-(Ala)_3_ heptapeptide. Following HPLC purification of diastereomers, structures of the two peptide forms were determined using X-ray crystallography and their structural characteristics were examined. The alanine residues on either side of the trishomocubane amino acid adopt a 3_10_-helical conformation and the cubane itself, in either diastereomeric form, is capable of causing a β-turn between these two short sections of helix. This trishomocubane amino acid serves as a novel covalent method for inducing β-turns in peptide chains and could potentially be employed with other, non-alanine, amino acids in an effort to generate β-sheet-type secondary structures.

**Figure 11.**
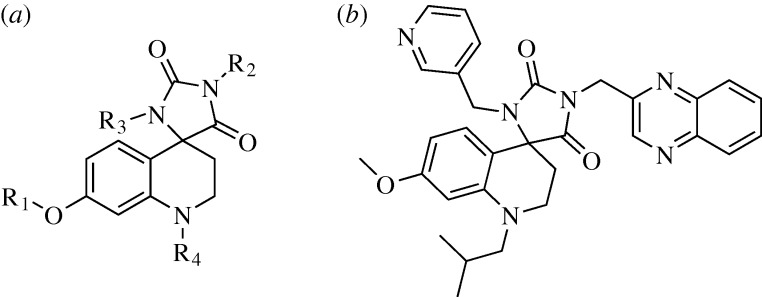
(*a*) Generic spyrocycle scaffold for β-turn mimicry. (*b*) Optimal MC4R agonist developed.

A common theme among turn inducing scaffolds is the use of spirocycles, owing to their ability to mimic the orientation of bonds at the α-carbon of an amino acid involved in a β-turn. Li recently made use of a non-peptidic spyrocycle (figure 11*a*) to mimic agonists of MC4R, a melanocortin family G-protein-coupled receptor known to bind peptides in a β-turn conformation (Webb *et al.* 2007). Interestingly, this scaffold was designed using cluster analysis principles pioneered by Garland & Dean (1999), which allow for the rapid searching of structural databases to locate scaffolds that position atoms in a similar manner to a peptidic β-turn. Following combinatorial exploration, an inhibitor with an EC_50_ of 0.7 μM versus MC4R was developed (figure 11*b*). Several of the compounds also showed encouraging selectivity for MC4R relative to other melanocortin and β-turn binding receptors, suggesting that the scaffold might eventually evolve into a highly selective binding agent.

One particularly well-investigated system involving a β-turn is somatostatin, a decapeptide hormone with a critical binding region comprising Phe-Trp-Lys-Thr in a β-turn motif. This hormone, which downregulates the release of numerous other hormones, is of interest for drug intervention. However, many turn mimetics do not mimic amino acid side-chain functionality, rendering them less useful in this system, where the turn is also the recognition sequence.

**Figure 12.**
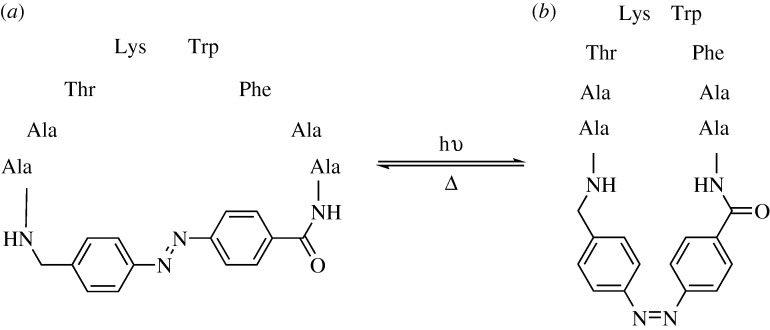
(*a*) An azobenzene turn inducer as a *trans*-isomer. The attached residues are spread out. (*b*) An azobenzene turn inducer as a *cis-*isomer. The attached residues form a β-turn.

Ulysse & Chmielewski (2006) have bypassed this problem with the construction of a cyclic peptide employing an azobenzene monomer opposite the tetrapeptide binding region. This allows both for the instigation of turn formation at the critical four residues and for light-modulated activation or deactivation of the peptide (figure 12).

When tested for binding against the somatotropin release-inhibiting factor receptor, a natural somatostatin target, the β-turn inducing *cis*-isomer was found to have a *K*_i_ of 1.0±0.1 μM, modestly improved relative to the 2.6±0.2 μM of the *trans*-isomer. Both isomers showed a *K*_i_ superior to that of a linear peptide analogue (*K*_i_=16±2 μM). This encouraging result suggests that azobenzene can not only act as a β-turn for attached peptides, but also modulate drug activity.

**Figure 13.**
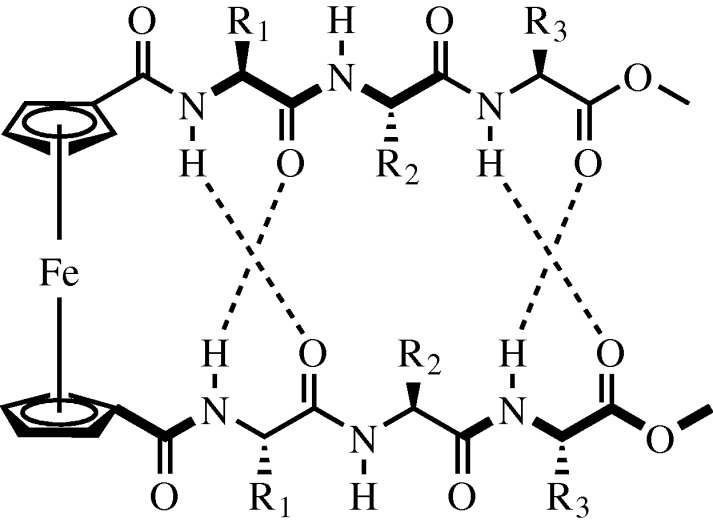
The use of a ferrocine tethered between two cyclopentadienyl terminated amino acid chains that feature a Gly-X-X motif allowing for β-sheet formation with cross-strand hydrogen bonds.

While novel developments in covalent methods for linking peptide chains to form stable β-sheet structures have been discussed, there has also been recent progress in non-covalent methods for orienting peptide strands. The Kraatz group have used ferrocene to coordinate two cyclopentadienyl rings attached to separate tripeptide strands (Chowdhury *et al.* 2008). Unlike previous efforts, a glycine residue was chosen at position R_1_ on each strand (figure 13). This selection took advantage of the high degree of phi and psi angles accessible to the glycine backbone to help facilitate cross-peptide-strand hydrogen bonding.

This work represents a significant advance over previous ferrocene amino acid conjugates where either peptide monomers (Henrick *et al.* 1996) or dimers (Moriuchi *et al.* 2001) have been used. Other research into this area has focused on the attachment of the peptides to the cyclopentadienyl core to generate either ‘parallel’ or ‘anti-parallel’ strands (Barisic *et al.* 2004; Chowdhury *et al.* 2005). The most recent report from the Kraatz group detailed the generation of β-helices (Chowdhury *et al.* 2006) and these efforts have now been extended to β-sheets. In addition, unlike some previous reports using both covalent and non-covalent coordination methods, researchers have now been able to maintain the β-sheet conformation beyond two residues, demonstrating a more complete β-sheet.

Another recent advance in the area of metal-chelation-driven assembly comes from the Breit group (Laungani *et al.* 2008) where platinum or rhodium was used to coordinate two diphenylphosphine ligands tethered to short peptide fragments. In several reports, the Breit group detailed the use of DNA A–T base pairing to discover new catalysts for the hydroformylation of terminal alkenes (Breit & Seiche 2003; Waloch *et al.* 2007). They have also described the discovery of self-assembled bi-dentate ligands for ruthenium-catalysed anti-Markovnikov hydration of terminal alkenes (Chevallier & Breit 2006) as well as for asymmetric rhodium-catalysed hydrogenation (Weis *et al.* 2006). More recently, they reported the attachment of one amino acid to their phosphine-based ligand binding compounds to impart greater catalyst specificity (Laungani & Breit 2008). All of these reports have hinged upon hydrogen bonding between two ligands of the bi-dentate metal binding pair.

**Figure 14.**
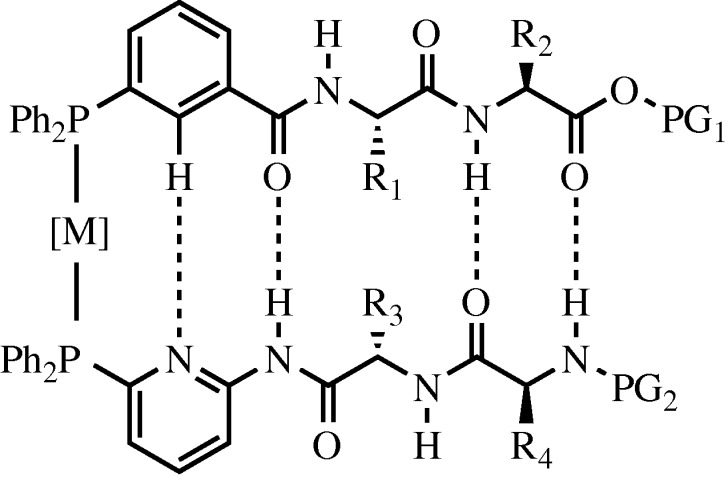
Metal tethered phosphine-based turn mimetic developed by Laungani *et al.* (2008).

This work has now been further extended to incorporate two amino acids along with a pyridine or phenyl ring, which are capable of intermolecular hydrogen bonding when properly aligned by metal chelation (figure 14). Prior to this work Schneider & Kelly (1995) chelated copper to a single peptide strand to help induce a β-sheet-type structure, and more recently Kruppa *et al.* (2005) used a peptide strand appended with zinc to coordinate a second peptide strand and form a β-sheet structure. The current approach used by Laungani *et al.* (2008) relies on a three-component system where the phenyl–peptide, pyridinyl–peptide and metal come together in a combinatorial fashion. This self-assembly was used to discover a novel catalyst for asymmetric rhodium-catalysed hydroformylation. Additionally, this process could easily be adapted to a dynamic combinatorial library (Rozenman *et al.* 2007) directed at the discovery of novel β-sheet protein mimetics.

## 4. Coil-based structures

Thus far, we have considered attempts to mimic only the most regularly encountered secondary structural motifs exhibited by biomacromolecules. However, some proteins participate in protein–protein interactions via so-called ‘random coil’ sequences. Such conformations are not at all random, however, and are ordered for any given system. While considered less systematically than other peptide structures, mimicry of such random coils can prove very promising. The emerging importance of the second mitochondrial activator of caspases (Smac) helps to exemplify this promise.

Apoptosis is a highly regulated cellular process that is at the centre of essentially all types of cancer. X-linked inhibitor of apoptosis (XIAP) is one of a family of IAP proteins that is known to be an inhibitor of apoptosis via its interaction with caspases. The specific interaction between the baculoviral IAP repeat (BIR) domain of XIAP and caspase-9 disrupts the ability of the caspase to adopt its active homodimeric conformation (Shiozaki *et al.* 2003). This disruption is conferred by the interaction of the BIR domain with the four *N*-terminal residues (Ala-Thr-Pro-Phe) of caspase-9. To alleviate this suppression, the second mitochondrial activator of caspases (Smac) is released from the mitochondria upon pro-apoptotic activation and, with its own four *N*-terminal residues (Ala-Val-Pro-Iso (AVPI)), competes for binding of the BIR domain of XIAP, releasing bound caspase-9 (Srinivasula *et al.* 2001). Based upon this information, Zobel *et al.* (2006) proposed that Smac AVPI mimetics would likely elicit a pro-apoptotic response in cells.

**Figure 15.**
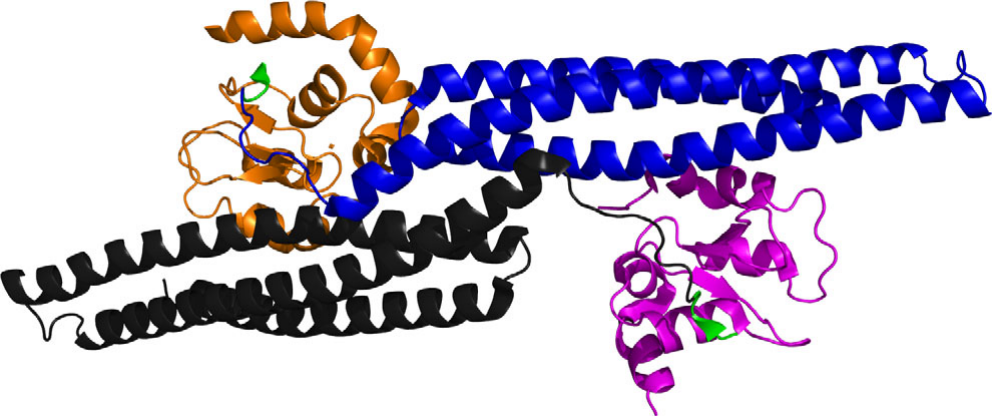
X-ray crystal structure of Smac (blue/black) bound to XIAP-BIR domain (orange/magenta), AVPI residues of Smac are shown in green (pdb 1G73; Wu *et al.* 2000).

**Figure 16.**
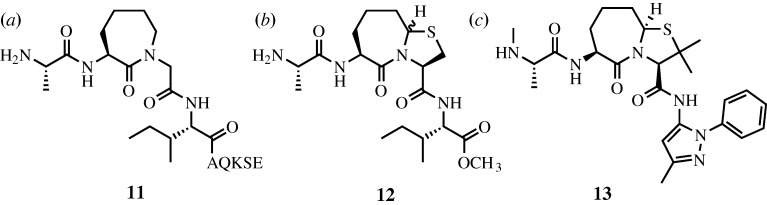
Structures of Zobel *et al.* (2006) compounds. Compounds (*a*) **11**, (*b*) **12** and (*c*) **13** depicting the evolution of Smac mimetics from more to less peptidic compounds.

**Figure 17.**
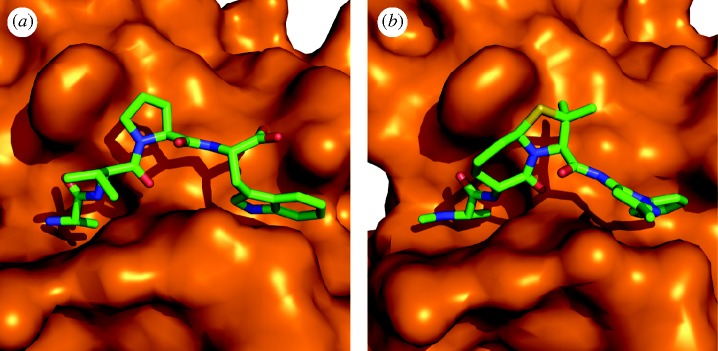
Initial lead compounds from the Deshayes group: (*a*) AVPW and (*b*) the final inhibitor **13**, both bound to a BIR domain as found in XIAP.

The four terminal residues of Smac are known to exist in a random coil conformation based upon X-ray crystallographic results (figure 15; Wu *et al.* 2000; Vucic *et al.* 2005; Zobel *et al.* 2006). In previous work, the Deshayes group had also determined via phage display that the affinity of a peptidic 4-mer for the BIR domain was increased if the sequence was altered to Ala-Val-Pro-Trp (AVPW; Franklin *et al.* 2003). Zobel *et al.* (2006) solved the X-ray crystal structure of AVPW bound to the BIR domain and found that it adopted a coil conformation highly similar to that of larger Smac peptides (RMSD C*α*=0.115 Å). However, AVPW has no biological activity and consequently a less peptidic compound was sought that would maintain the contacts made by AVPW. Progressively less peptidic compounds were screened where **11** (figure 16*a*) was found to inhibit the BIR–Smac interaction with an inhibition constant (*K*_i_) of 15 μM. This then was further modified to **12** (*K*_i_=1 μM), and finally **13**, which disrupted the interaction with 20-fold higher potency (*K*_i_=50 nM). X-ray crystallographic analysis demonstrated that **13** bound the BIR domain in the same conformation as the AVPW peptide (figure 17). However, unlike AVPW, **13** was found to have activity *in vitro* and was also capable of protecting both breast cancer and melanoma cells by causing increased apoptosis.

**Figure 18.**
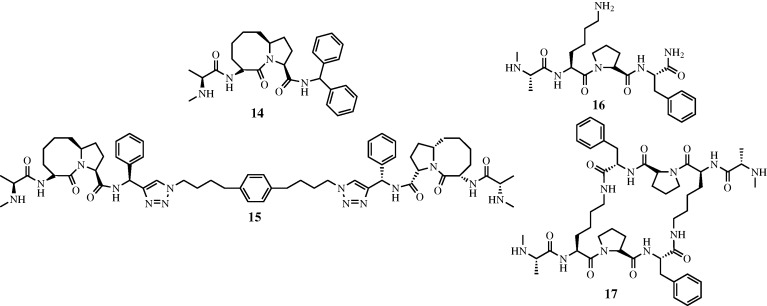
Smac mimetics from the Wang group, where dimerization of Smac mimetics has been shown to be an effective means of increasing compound potency and efficacy.

The Wang group has expanded upon this area of mimetic research by generating both linear (Sun *et al.* 2007) and cyclic (Nikolovska-Coleska *et al.* 2008) bivalent Smac mimetics that bind to BIR domains, as in XIAP. In the case of their linear mimetics (figure 17) Sun and co-workers were able to realize a 300-fold increase in binding affinity against targets containing both a BIR2 and a BIR3 domain when compared with the monovalent inhibitor counterparts (IC_50_=1.39 nM versus 417 nM). In the case of their more constrained cyclic bivalent derivative, a 22-fold improved activity over the monovalent version is observed (*K*_i_=4 nM versus 86 nM; figure 18). Additionally, a co-crystal structure of bivalent **17** with the BIR3 domain of XIAP depicts **17** cross-linking two monomeric units of XIAP, helping to explain its potency. Ultimately, in both the linear and the cyclic systems, these compounds proved effective modulators of XIAP function both *in vitro* and in cell-based models for multiple cancers and provide compelling examples for the use of multivalent inhibitors.

## 5. Conclusions

We have herein highlighted several advances in the area of protein secondary structure mimetics. In particular, we have focused on molecules that are able to project functionality in the same fashion as a protein and have validated activity against biological targets. The drive to expand libraries and improve the pharmacological properties of compounds suggests that the field is ripe for ground-breaking mimetic scaffolds targeting the important secondary structure elements involved in so many key biological processes.
